# Mystery Solved: Why Smoke Extract Worsens Disease in Smokers with Crohn’s Disease and Not Ulcerative Colitis? Gut MAP!

**DOI:** 10.3390/microorganisms8050666

**Published:** 2020-05-02

**Authors:** Dania AlQasrawi, Latifa S. Abdelli, Saleh A. Naser

**Affiliations:** Division of Molecular Microbiology, Burnett School of Biomedical Sciences, College of Medicine, University of Central Florida, Orlando, FL 32816, USA; daniaqasrawi@knights.ucf.edu (D.A.); latifa.abdelli@ucf.edu (L.S.A.)

**Keywords:** Crohn’s Disease, nicotine, MAP, macrophages, α7nAChR

## Abstract

Cigarette smoke (CS) exacerbates symptoms in Crohn’s disease (CD) patients while protecting those with ulcerative colitis (UC). CD has been associated with immuno-dysregulation, mucosal dysfunction, and infection. Among the CD-debated pathogens are *Mycobacterium avium* subsp. *paratuberculosis* (MAP), adherent invasive *Escherichia coli* (AIEC), and *Klebsiella pneumoniae*. The mechanism of how CS modulates nicotinic acetylcholine receptor-α7 (α7nAChR) and elicits inflammatory response in CD-like macrophages is unknown. Here, we investigated the effect of CS/nicotine on macrophages infected with CD-associated pathogens. We measured apoptosis, bacterial viability, macrophage polarization, and gene expression/cytokine levels involved in macrophage response to nicotine/CS extracts from Havana-Leave extract (HLE-nicotine rich) and germplasm line of Maryland tobacco (LAMD-nicotine less). Nicotine (4 µg/mL) and HLE extracts (0.18%) significantly favored anti-inflammatory response in macrophages (increased CD-206 (M2) and IL-10, and decreased M1/M2 ratio; *p* < 0.05). While macrophages infected with MAP or treated with LPS promoted pro-inflammatory response. Further treatment of these macrophages with nicotine or HLE extracts caused higher inflammatory response (increased iNOS (M1), TNF-α, IL-6, and M1/M2 ratio, *p* < 0.05), increased MAP burden, and decreased apoptosis. Pre-conditioning macrophages with nicotine ahead of infection resulted in lower pro-inflammatory response. Blocking α7nAChR with an antagonist voided the effect of nicotine on macrophages. Overall, the study provides an insight toward understanding the contradictory effect of nicotine on Inflammatory Bowel Disease patients and about the mechanistic role of α7nAChR in modulation of macrophages in tobacco smokers.

## 1. Introduction

Cigarette smoke (CS) is considered an independent risk factor in the development of Crohn’s disease (CD) [1,2,3]. It is associated with exacerbated inflammatory relapses, postoperative recurrence, and poorer response to medical therapy [1,2,3]. A standard clinical practice for newly diagnosed CD patients is to quit CS to avoid the aforementioned debilitating consequences [4,5]. Generally, CS causes CD smokers to suffer from exacerbated inflammatory symptoms, severe phenotypical presentation of the disease, and in many cases leads to the patient’s hospitalization and poor long-term prognosis [6,7,8,9]. On the contrary, CS seems to be surprisingly protective in patients with ulcerative colitis (UC), which is largely known as the disease of the non-smokers [3,5]. Inflammatory symptoms in UC are significantly decreased when tobacco is consumed, while smoking cessation results in abrupt flares of inflammation and more frequent relapsing episodes in these patients [10]. UC patients with history of smoking usually acquire their disease few years after they stop smoking, suggesting that smoking protected them from acquiring the disease earlier [11]. Unlike UC, the etiology of CD has been vigorously researched and debated and now it is generally accepted that CD is associated with microbial infection, immune-dysregulation, and mucosal dysfunction. Among the most debated pathogens that have been studied for possible roles in CD pathogenesis are *Mycobacterium avium* subsp. *paratuberculosis* (MAP), adherent-invasive *Escherichia coli* (AIEC), and *Klebsiella pneumoniae* [12,13,14]. The mechanism of how CS ingredients induce pro-inflammatory or anti-inflammatory phenotypes in IBD remained unclear. This study is not about what causes CD; it is about understanding the mechanism involved in how CS affects macrophages infected with microorganisms associated with CD pathogenesis. The monocytic response against intracellular bacterial infection such as MAP is usually mediated by TLR2 receptor through the recruitment of cytoplasmic adaptor protein and activation of intracellular signaling molecules, which lead to phagosome maturation and synthesis of pro-inflammatory cytokines like TNF-α, IL-6, IL-8, IL-12, and IL-23 [15,16].

Nicotine, an addictive immunosuppressant ingredient in CS, is known to decrease the level of pro-inflammatory cytokines, inhibit dendritic cells, and prevent cell apoptosis [17,18]. Nicotine also decreases the production of cathelicidin, anti-microbial peptide, and inhibits formation of granuloma [19,20]. The concentration of nicotine in active cigarette smokers ranges between 4 and 72 ng/mL [21]. Although smokeless tobacco products, including e-cigarettes and smoking cessation nicotine-replacement products, are labeled as somewhat safe, the level of nicotine in the user’s blood can exceed the level achieved by cigarette smoking [22]. Tobacco-use is the leading cause of preventable disease and death in the United States [23]. Nearly half a million Americans die prematurely due to smoking or secondhand smoking exposure [23]. Another 16 million Americans live with serious illnesses caused by cigarette smoking [5]. Nicotine as a potent alkaloid is considered to be the most addictive and pharmacologically active substance among the many compounds present in tobacco products.

Limited studies have investigated how the innate immune response interplays in the overall inflammatory process involved in IBD and what effects nicotine has on macrophage recruitment, proliferation, and phenotypic response in CD and UC subsets. One clear link between innate immune cells and CS is the presence of a nicotinic acetylcholine receptor α7 (α7-aAChR) on monocytes and macrophages cell surface [24]. Experimental studies have demonstrated that nicotine inhibits pro-inflammatory cytokine release from in vitro monocytes-based cell culture [24]. However, this does not explain the conflicting effects of CS on UC and CD symptoms. Generally, monocytes respond to direct stimuli by differentiating into classical pro-inflammatory M1 cells or alternative anti-inflammatory M2 macrophages [25]. M1 releases IL-1b, MCP-1, iNOS, IL-6, IL-8, IL-12, IL-23, and TNF-α, while M2 macrophages release IL-10, IL-1Ra, and higher levels of arginase-1. The latter inhibits nitric oxide production and reduces inflammation [25]. This study is designed to study the mechanism of how CS or its most abundant and active ingredient, nicotine, modulates α7aAChR on macrophages infected with intracellular pathogens and elicit inflammatory response. Specifically, the study focused on investigating the effect of CS and nicotine on the cellular processes in THP-1 infected with MAP, or LPS derived from AIEC, mimicking CD smokers associated with bacterial infection.

## 2. Materials and Methods

### 2.1. Culture Conditions of THP-1 Macrophages

THP-1 monocytes (ATCC TIB-202) were cultured as described earlier [26]. Briefly, cells were maintained in a RPMI-1640 growth medium (Invitrogen, Carlsbad, CA, USA) containing 10% fetal bovine serum (FBS) (Invitrogen) and 0.09% β-mercaptoethanol and maintained in a humidified 5% CO_2_ incubator at 37 °C until they reached 80% confluency. Then, cells were activated by adding 50 ng/mL of phorbol 12-myristate 13-acetate (PMA) (Sigma-Aldrich, St. Louis, MO, USA) for 48 h. All experiments were performed in quadruplicates and done within 12 passages. 

### 2.2. Infection and Treatment of THP-1 Macrophages

*Infection:* activated THP-1 cells (M0 macrophages) were plated at a density of 3 × 10^5^ cells in 12 well plates. Cells were infected for 24 h at 37 °C in 5% CO_2_ with 1 × 10^7^ CFU/mL of MAP strain UCF4 (a clinical strain isolated from CD patient), *Mycobacterium tuberculosis* ATCC HR237, or *Klebsiella pneumoniae* ATCC13883. Bacterial Lipopolysaccharide (LPS) from *Escherichia coli* strain O111.B4 (Sigma Aldrich, 1 µg/mL) and uninfected cells were used as a control. 

*Treatment with CS extracts and nicotine:* activated THP-1 macrophages were treated with nicotine (0 to 4 µg/mL, Sigma-Aldrich), 0.18% CS extracts from HLE-Petit Havana (tobacco leaves rich with nicotine), or 0.18% germplasm line of Maryland tobacco (LAMD, tobacco leaves with nicotine-less) for 24 h at 37 °C in 5% CO_2_. HLE and LAMD tobacco leaves were generously provided by Professor Henry Daniel (University of Pennsylvania).

*Nicotine Receptor Inhibitor:* To determine the role of α7nAchR in the cellular uptake of CS and nicotine, macrophages were treated with mecamylamine (MEC), an α7nAChR antagonist (Fisher Scientific). Macrophages were pretreated with 0, 25, 50 and 75 µM of MEC at room temperature for 30 min prior to nicotine or CS treatments. All experiments were performed in quadruplicates.

### 2.3. Measurement of Relative Gene Expression Using RT-PCR

*RNA Isolation*: cell pellets were collected in 2 mL-microcentrifuge and suspended in 500 µL of TRIzol^®^ reagent (Invitrogen). A total of 125 µL of chloroform was added to each tube, mixed, and incubated at room temperature for 5 min. Following centrifugation at 10,000 rpm for 5 min, the aqueous phase was transferred to a new tube and mixed with 275 µL isopropanol. Following centrifugation at 14,000 rpm and 4 °C for 15 min, RNA pellets were isolated, washed in 500 µL of 75% ethanol, air-dried, and then dissolved in 15 µL of Tris-EDTA (TE) buffer. RNA concentration and purity were analyzed using Nonodrop OneC (ThermoFisher) and stored at –80 °C until further use.

*cDNA Synthesis:* Reverse transcription reaction was carried out by adding 800 ng of RNA in 0.25-mL microtubes containing 20 µL of PCR reaction, 4 µL iScript™ Reverse transcription (Bio-Rad^®^, Hercules, CA, USA) and up to 0.2 mL RNAse-free water. The reaction was performed using MyGene Series Peltier Thermal Cycler under the following conditions: 5 min at 25 °C, 20 min at 46 °C, and 1 min at 95 °C. 

*RT-PCR:* Gene expression analysis was performed as described earlier [27]. Briefly, a total volume of 20 µL reaction mixture containing 1 µL of cDNA (30 ng/µL), 10 µL of Fast SYBR Green Mastermix (Thermo Fisher Scientific, Waltham, MA, USA), 1 µL of either *iNOS, CD206, IL-6, TNF-α*, or *IL-10,* PrimePCR SYBER Green Assay mixes (Bio-Rad^®^, California, USA), and 8 µL of RNAse-free water were prepared in a 96-well Microamp RT-PCR reaction plate and analyzed using 7500 Fast Real-Time PCR System (Applied Biosystems, Foster City, CA, United States). Housekeeping GAPDH primer (Bio-Rad^®^, California, USA) was used to obtain baseline CT readings. Relative mRNA expression was calculated by using the equation 2^(−∆CT)^, where ∆CT = [(Sample RT-PCR CT value) − (GAPDH CT baseline value)] × 1000. 

### 2.4. Measurement of Cytokines and Cell Surface Markers

For cell surface markers, cell pellets were incubated with ice RIPA buffer (Thermo-Fisher, Cat# 89901, Massachusetts, USA) for 15 min, then centrifuged at 14,000 rpm for 20 min. Supernatant containing cell lysates were analyzed using ELISA kits: iNOS (LS-Bio; Washington, USA, Cat# LS-F39142), CD206 (Sigma-Aldrich; Cat# RAB1178, Missouri, USA) following manufacturer’s instructions. Cell culture supernatants were used to measure cytokine levels: IL-6 (LS-Bio; Cat# LS-F9754, Washington, USA), TNF-α (LS-Bio; Cat# EH3TNFA, Washington, USA), and IL-10 (Sigma-Aldrich; Cat# KHC0101, Missouri, USA). All ELISA experiments were done in triplicates. 

### 2.5. Bacterial Viability Assay 

Following the infection, macrophages were washed twice with PBS to remove extracellular bacteria, then lysed using cold RIPA buffer (Thermo-Fisher, Cat# 89901, Massachusetts, USA) for 15 min. Cell lysates were centrifuged at 14,000 rpm for 20 min and the supernatant was collected to perform bacterial viability using the Live/Dead Baclight Bacterial Viability Kit (Molecular Probes, Inc., Eugene, OR, USA), as described earlier [26]. All viability experiments were performed in triplicates.

### 2.6. Measurement of Macrophage Apoptosis

In order to examine the apoptotic pathway induced by nicotine, caspase-3 colorimetric assay (Abcam, Cambridge, UK) was used as described earlier [26]. Briefly, following infection and treatment, THP-1 cells were washed twice with PBS, collected and lysed using chilled Cell Lysis Buffer. Total protein levels were analyzed using DEVD-pNA as a caspase-3-specific substrate. After incubation at 37 °C for 1–2 h, absorbance was measured at 400–405 *nm* in a SpectraMax i3x plate reader. All apoptosis experiments were done in duplicate (*n* = 2) with each sample loaded in triplicate and the average was calculated.

### 2.7. Statistical Analysis

All data collected in this study were pre-tested for normal distribution using the Kolmogorov–Smirnov normality test followed by Unpaired Two-tailed *t* test. *p* < 0.05 and a 95% confidence interval (CI) were used for the assessment of differences in all experiments. Data were presented as Mean ± SD. *p*-values <0.001 were also mentioned when achieved.

## 3. Results

### 3.1. Determination of Ingredient(s) in CS Extracts Active in Modulating Macrophages Response

In order to determine the active ingredient(s) in tobacco leaves that modulate inflammatory response in macrophages, we measured M1 (*iNOS*) and M2 (*CD206*) markers, *IL-6, TNF-α,* and *IL-10* expression in PMA-activated THP-1, and macrophages infected with MAP following a treatment with CS extracts from HLE-Petit Havana (nicotine-rich) and LAMD (nicotine-less). We estimated that 0.18% of HLE-Petit Havana contains 4 µg/mL nicotine [28]. Accordingly, we used 0.18% HLE-Petit Havana extracts in parallel to pure nicotine (4 µg/mL) and 0.18% of LAMD tobacco. Experiments were also performed with LPS-derived from AIEC. MTB was included as control.

### 3.2. Uninfected Cells

**Figure 1:**
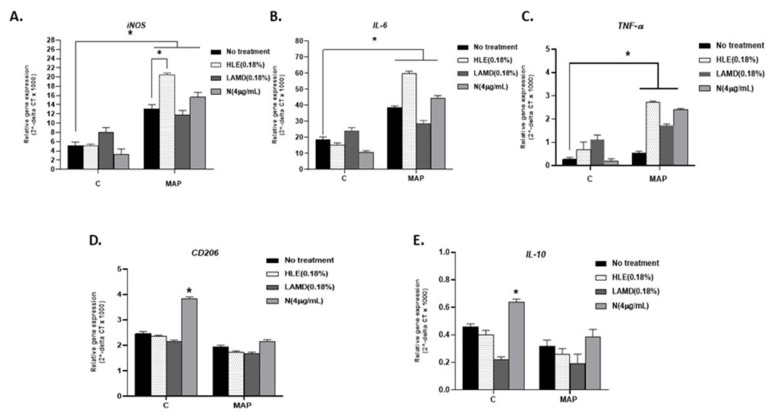
Effect of cigarette smoke (CS) and pure nicotine on *Mycobacterium avium* subsp. *paratuberculosis* (MAP)-infected macrophages. Figure 1 depicts RT-PCR results for iNOS (**A**), IL-6 (**B**), TNF-α (**C**), CD206 (**D**), and IL-10 (**E**) gene expression, respectively, for THP-1 phorbol 12-myristate 13-acetate (PMA) differentiated macrophages infected with MAP and then treated with 0.18% of Havana-Leave extract (HLE)-Petit Havana (with nicotine), 0.18% of germplasm line of Maryland tobacco (LAMD) (nicotine low), and nicotine (4 µg/mL). Data is shown as Mean ± SD. Independent experiments were done twice (*n* = 2) with each sample repeated three times. * *p* < 0.05.

Although the effect of CS extracts from both HLE and LAMD on *iNOS*, *CD206, IL-6,* and *TNF-α* was mostly similar (Figure 1A–D), expression of *IL-10* was significantly low in LAMD-treated cells (0.22 ± 0.021; *p* < 0.05) compared to HLE-treated cells (0.40 ± 0.033; *p* < 0.05; Figure 1E). Likewise, the effect of nicotine on *iNOS, IL-6*, and *TNF-α* was not significant, but it increased the expression of *CD206* (3.84 ± 0.06 vs. untreated cells 2.46 ± 0.08; *p* < 0.05; Figure 1D) and *IL-10* (0.64 ± 0.020 vs. untreated 0.46 ± 0.020; *p* < 0.05; Figure 1E).

### 3.3. Infected Cells

#### 3.3.1. In Absence of CS Extracts and Nicotine

Infection of macrophages with MAP increased *iNOS* expression (13.09 ± 0.94 vs. 5.11 ± 0.8 in uninflected cells), *IL-6* (38.43 ± 1.2 vs. 18.5 ± 1.8 in uninfected cells), and *TNF-α* (0.54 ± 0.075 vs. 0.27 ± 0.08 in uninflected cells). 

#### 3.3.2. In Presence of CS Extracts and Nicotine

We also evaluated the effect of CS extracts from both HLE and LAMD, as well as nicotine on macrophages infected with MAP, and the outcome was surprisingly unexpected (Figure 1). CS extracts from HLE and nicotine both further increased *iNOS* expression in MAP-infected macrophages compared to CS extracts from LAMD and uninfected cells (HLE: 20.49 ± 0.4; nicotine: 15.64 ± 1.05; LAMD: 11.76 ± 1; and uninfected: 13.09 ± 0.94; *p* < 0.05; Figure 1A). The outcome was validated by a measurement of corresponding cytokines. Expression of *IL-6* and *TNF-α* increased in MAP-infected macrophages and subsequently treated with CS extracts of HLE or nicotine compared to CS extracts of LAMD and untreated cells (HLE: 59.8 ± 1.4; nicotine: 44.5 ± 1.5; LAMD: 28.5 ± 2; and uninfected: 18.5 ± 1.8; *p* < 0.05 for *IL-6*) and (HLE: 2.7 ± 0.04; nicotine: 2.4 ± 0.05; LAMD: 1.7 ± 0.07; and uninfected: 0.27 ± 0.08; *p* < 0.05 for *TNF-α*; Figure 1B–C). The effect on CD206 and *IL-10* expression was not significant (Figure 1D,E).

### 3.4. Effect of Nicotine on Macrophages Response is Dose-Dependent 

**Figure 2:**
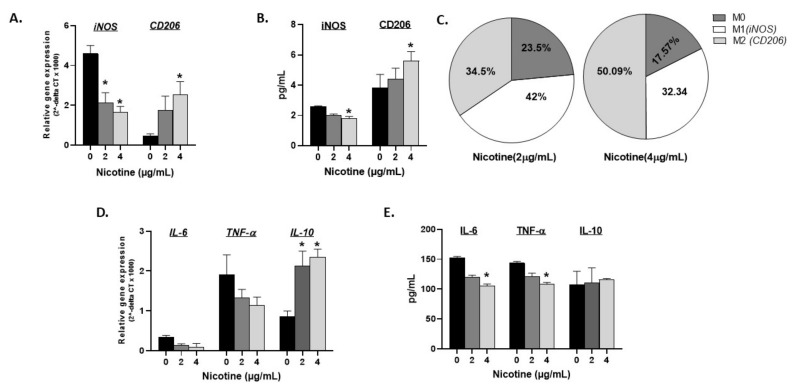
Nicotine induces dose dependent M2 macrophage polarization in vitro. Activated THP-1 were treated with ascending concentrations of nicotine and evaluated at gene expression and protein levels for iNOS, CD206, IL-6, TNF-α, and IL-10 using RT-PCR (**A**, **D**) and ELISA (**B**, **E**), respectively. (**C**): Overall gene expression distribution for M1, M2, and activated M0 macrophages in presence of 2 and 4 µg/mL of nicotine. All experiments were performed in triplicates. * *p* < 0.05.

The effect of nicotine dosage on macrophage polarization and response was investigated. Activated THP-1 cells were treated with ascending concentrations of nicotine (0, 2, and 4 µg/mL) for 24 h followed by RT-PCR and ELISA evaluation of the expression and production of iNOS and CD206 markers. As shown in Figure 2 (A: gene expression, and B: protein levels), iNOS (M1) gradually decreased and CD206 (M2) increased in the dose-dependency of nicotine (*p* < 0.05). Based on these two markers’ gene expression, the percentage of M1, M2, and undifferentiated M0 macrophages were calculated in presence and absence of nicotine. Untreated cells were differentiated into M1:M2 macrophages with a ratio of 9:1 of M1:M2 (calculated from black bars in Figure 2A). Figure 2C depicts how nicotine treatment shifts this ratio. In fact, nicotine treatment at 2 µg/mL starts to shift polarization with a ratio of 42% M1 vs. 34.5% M2 and 23.5% M0 cells while 4 µg/mL nicotine further increased M2 macrophages to 50.09% against 32.34% M1 and around 17.57% undetermined M0 cells. Gene expression and protein levels of IL-6, TNF-α, and IL-10 were also illustrated in Figure 2D,E, respectively. Data indicate that an ascending concentration of nicotine correlates with decreased IL-6 and TNF-α gene and protein expression, which is reflective of the decreased M1 polarization marker iNOS. Accordingly, anti-inflammatory IL-10 gene expression and not protein significantly increased with 2 and 4 µg/mL nicotine treatment. 

### 3.5. Effect of Nicotine on Macrophages Is Mediated through α7nAChRs

#### 3.5.1. In Absence of Infection

**Figure 3:**
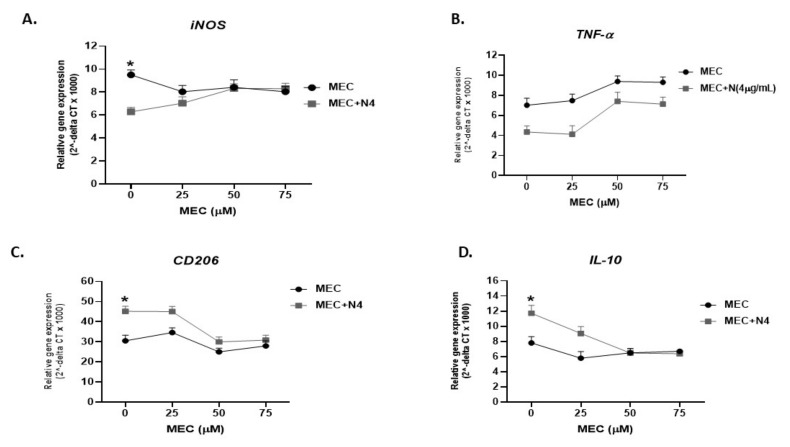
Mecamylamine (MEC) abrogates the immunosuppressive effect of nicotine in vitro. THP-1 PMA differentiated macrophages were incubated with 4 µg/mL of nicotine after pretreatment with (0, 25, 50, and 75 µM mecamylamine (MEC)). iNOS (**A**), CD206 (**C**), TNF-α (**B**), and IL-10 (**D**) gene expression was analyzed by RT-PCR, respectively. All experiments were performed in triplicates. * *p* < 0.05.

To determine if nicotine modulates macrophages and shifts polarization to anti-inflammatory response is mediated through α7nAChRs, we blocked nAchR with mecamylamine (MEC), an nAchR antagonist. Specifically, activated THP-1 macrophages were pretreated with 0, 25, 50 and 75 µM MEC, followed by 4 µg/mL of nicotine. As shown in Figure 3A, in absence of MEC, nicotine significantly decreased the *iNOS* level (6.25 ± 0.32 compared to 9.5 ± 0.25 in macrophages not treated with nicotine; *p* < 0.05). Preconditioning cells with MEC (25 to 75 µM) gradually neutralized the effect of nicotine on *iNOS* and reached its optimum effect at 50 µM (Figure 3A). A similar effect was also observed on *TNF-α* expression (Figure 3B). Likewise, pretreatment of macrophages with MEC has altered the effect of nicotine on *CD206* (MEC 50 µM + nicotine 4 µg/mL: 29.97 ± 2.45 vs. MEC 50 µM: 25 ± 1.81 vs. MEC 0 µM control: 30.5 ± 2.82; *p* < 0.05), Figure 3C, and *IL-10* (MEC 50 µM + nicotine: 6.47 ± 0.56 vs. MEC 50 µM: 6.5 ± 0.56 vs. MEC 0 µM control: 7.8 ± 0.84; *p* < 0.05), Figure 3D.

#### 3.5.2. During Infection

**Figure 4:**
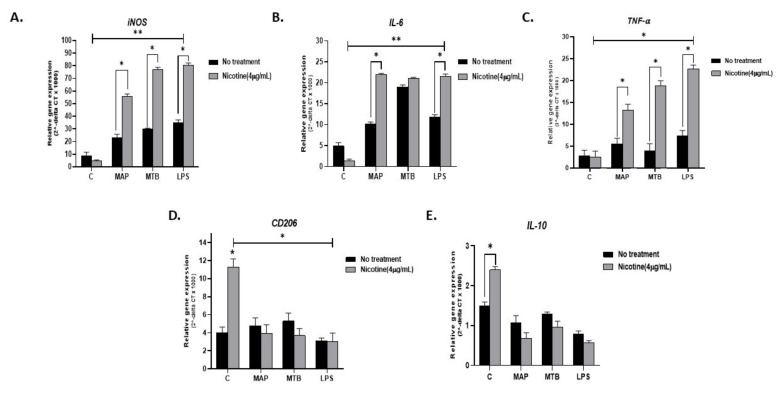
Effect of nicotine on macrophage response and infection. THP-1 PMA differentiated macrophages were infected with MAP and MTB, or treated with LPS for 24 h, then treated with nicotine (4 µg/mL). Expression of iNOS (**A**), IL-6 (**B**), TNF-α (**C**), CD206 (**D**), and IL-10 (**E**) was measured by RT-PCR. All experiments were performed in triplicates. MAP: *mycobacterium avium paratuberculosis*. MTB: mycobacterium tuberculosis. LPS: lipopolysaccharide derived from *Escherichia coli* ATCC 8739. * *p* < 0.05, ** *p* < 0.001.

To explain why smokers with CD experience heightened inflammatory episodes compared to non-smoking CD patients or UC patients, we infected activated THP-1 with MAP for 24 h followed by treatment with 4 µg/mL nicotine. MTB and LPS were also used as controls. As expected, all infections resulted in a significant increase in pro-inflammatory status as evidenced by increased expression of *iNOS, IL-6*, and *TNF-α* (Figure 4A–C). Interestingly, adding nicotine treatment to infected macrophages almost doubled *iNOS* readings across all infections (MAP + nicotine: 55.7 ± 2.02 vs. MAP alone: 22.94 ± 2.87), (MTB + nicotine: 77.15 ± 1.6 vs. MTB alone: 29.81 ± 0.81. *p* < 0.05), or (LPS + nicotine: 80.39 ± 1.83 vs. LPS alone: 35.16 ± 2.04; *p* < 0.05). The significance effect was also observed when compared with untreated and uninfected control cells; *p* < 0.001. A similar trend was observed for *IL-6* and *TNF-α* gene expression (Figure 4B,C). It is worth noting that the effect of MTB on *IL-6* was far superior to that of MAP or LPS (MTB: 19 ± 0.5 vs. MAP 10.14 ± 0.5 vs. LPS 11.8 ± 0.6. *p* < 0.05), Figure 4B. However, the increase in *IL-6* level following treatment with nicotine of infected macrophages was similar among MAP, MTB, and LPS. There was a significant decrease in the expression of the anti-inflammatory CD206 marker following nicotine treatment of infected macrophages (*p* < 0.05), Figure 4D. IL-10 showed a clear decrease in expression following nicotine treatment of infected macrophages, but it was not statistically significant (Figure 4E).

**Figure 5:**
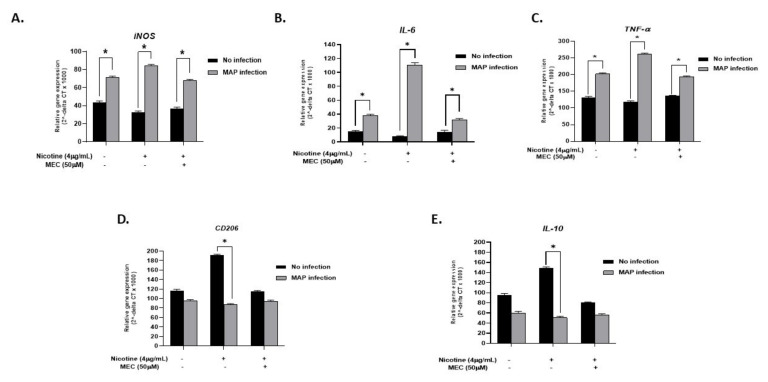
Interplay between nicotine, α7nAChR, and mecamylamine (MEC, an nAchR antagonist) in macrophages. THP-1 PMA differentiated macrophages were infected with MAP and then incubated with 4 µg/mL of nicotine after pretreatment with 50 µM mecamylamine (MEC). Expression of iNOS (**A**), IL-6 (**B**), TNF-α (**C**), CD206 (**D**), and IL-10 (**E**) was analyzed by RT-PCR. All experiments were performed in triplicates. * *p* < 0.05.

To validate that the effect of nicotine on infected cells is mediated through α7nAChRs, we blocked nAchR in macrophages with MEC. Treatment with MEC seems to cancel the nicotine effect with *iNOS* expression levels more aligned with those found with MAP infection only (MAP + MEC + N: 67.9 ± 1.42 vs. MAP: 71.22 ± 1.54. *p* < 0.05, Figure 5A). Pro-inflammatory cytokines *IL-6* and *TNF-α* also reflected the *iNOS* results, with MAP infection enhancing their expression, nicotine to further increase their expression, and MEC to cancel the nicotine effect back to a MAP inflammatory status (Figure 5B,C for *IL-6* and *TNF-α,* respectively). Anti-inflammatory marker *CD206* and *IL-10* cytokine also demonstrated a significant increase in gene expression upon nicotine treatment of uninfected macrophages (nicotine: 191.16 ± 2.4 vs. control: 116.81 ± 2.6. *p* < 0.05) for CD206. Interestingly, MEC pre-conditioning prevented nicotine-induced *CD206* and *IL-10* gene expression; (114.5 ± 2.2 vs. 191.16 ± 2.4. *p* < 0.05) for CD206 and (79.8 ± 2.1 vs. 148.53 ± 3.2. *p* < 0.05) for *IL-10* (Figure 5D,E, respectively).

### 3.6. Pre-Conditioning Macrophages with Nicotine Protects against Infection-Induced Inflammation

#### Prior to Infection

**Figure 6:**
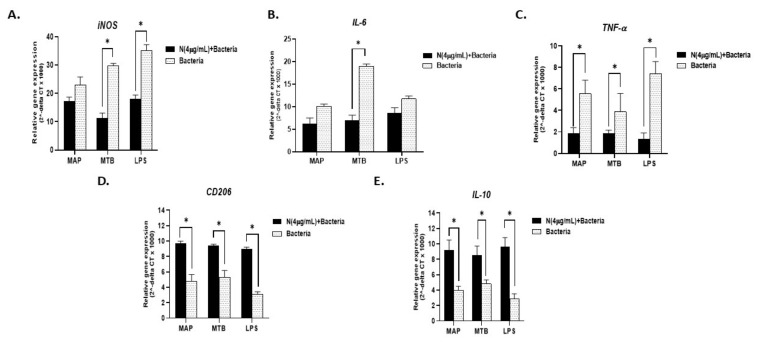
Effect of nicotine preconditioning on infected macrophages. RT-PCR results for expression of iNOS, IL-6, TNF-α, CD206, and IL-10, (**A**–**E**, respectively). THP-1 PMA differentiated macrophages treated with 0 and 4 µg/mL of nicotine for 24 h then infected with MAP, MTB, or incubated with LPS for further 24 h. All experiments were performed in triplicates. * *p* < 0.05. MAP: *mycobacterium avium paratuberculosis*. MTB: mycobacterium tuberculosis. LPS: lipopolysaccharide derived from *Escherichia coli* ATCC 8739.

Due to the bivalent effect of nicotine in IBD, we wondered whether the timing of infection in IBD smokers could determine the direction of the inflammatory shift. To address that, we evaluated the inflammatory profile of macrophages pre-conditioned with nicotine before infection with MAP (nicotine exposure prior infection) versus macrophages that were infected with MAP. MTB and LPS were used as controls. As shown in Figure 6A, the *iNOS* level was significantly lower in cells preconditioned with nicotine prior to infection (MAP: 22.9 ± 2.8 vs. nicotine + MAP: 17.17 ± 1.5. *p* < 0.05), (MTB: 29.8 ± 0.8 vs. nicotine + MTB: 11.35 ± 1.7. *p* < 0.05), or (LPS: 35.16 ± 2.04 vs. nicotine + LPS: 18.06 ± 1.4); *p* < 0.05). Similarly, *IL-6* and *TNF-α* gene expression results (Figure 6B,C, respectively) corroborated those of *iNOS* with the lowest values consistently reported for cells preconditioned with nicotine prior to infection. M2 cell surface marker CD206 and IL-10 cytokine, on the other end, showed their highest expression levels (*p* < 0.05) in cells pre-exposed to nicotine prior to infection (Figure 6D,E, respectively).

### 3.7. Nicotine Increases MAP Burden in Macrophages and Decreases Cellular Apoptosis

To determine MAP viability and cellular apoptosis in macrophages treated with nicotine prior and post infection, activated THP-1 cells were infected with MAP for 24 h, then treated with nicotine (4 µg/mL), and vice versa. As listed in Table 1, nicotine maintained viability and increased the burden of MAP and MTB regardless of the timing of exposure to nicotine prior or post infection. For MAP: nicotine + MAP 91.4 ± 5.5, MAP alone 69.4 ± 5.2, and MAP + nicotine 80.79 ± 4.8. For MTB: nicotine + MTB 92.5 ± 4.6, MTB alone 74.2 ± 3.1, and MTB + nicotine 85.2 ± 6.2. *K. pneumoniae* was used as a negative control and the viability was the minimum as expected.

**Figure 7:**
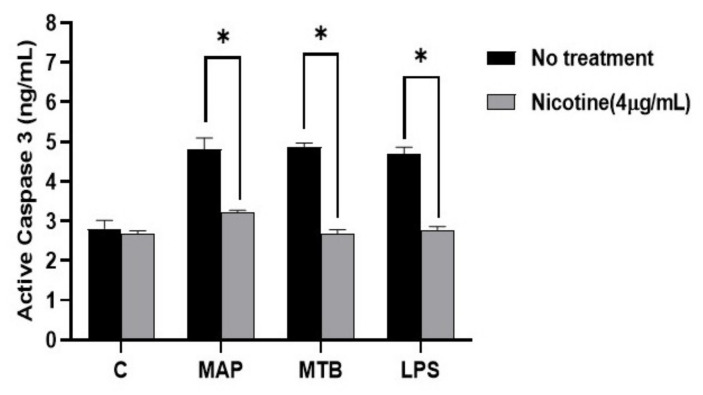
Effect of nicotine on apoptosis in macrophages post infection. Caspase-3 activity was measured in differentiated macrophages following bacterial infection and treatment with 4 µg/mL of nicotine. All experiments were performed in triplicates. * *p* < 0.05.

For apoptosis, active caspase-3 activity increased in MAP infected macrophages when compared to control, which was similar to macrophages infected with MTB or treated with LPS (Figure 7). The data also show that the activity of caspase-3 in macrophages was lower in nicotine treated macrophages after MAP infection (MAP: 4.81 ± 0.28 vs. MAP + nicotine: 3.22 ± 0.05. *p* < 0.05). A similar trend was also observed for MTB and LPS. 

## 4. Discussion

More studies are needed to elucidate the role of innate immune response in the overall inflammatory process in IBD smokers. Limited studies have investigated the effect of CS or nicotine on macrophage recruitment, proliferation, and consequent immune response among smokers with CD or UC. Extensive studies reported a strong association between microbial infection and CD pathogenesis; the opposite is true for UC [29]. Infection of macrophages with intracellular microorganisms, such as MAP or AIEC, CD-associated pathogens, leads to pro-inflammatory response and increase in M1/M2 ratio [30]. On the contrary, nicotine binds to α7nAChR on monocytes and the macrophages cell surface and inhibits pro-inflammatory cytokine release leading to a decrease in M1/M2 ratio [31,32]. Extensive studies have investigated the effect of CS in patients with tuberculosis. Most reports have demonstrated a strong link between smoking and increased susceptibility to tuberculosis (TB) infection [33]. For example, studies reported that TB smokers have higher levels of alveolar macrophages compared to non-smoker and former smokers with TB, which may explain the abnormal innate immune response in these patients [33,34]. Since there is strong homology between MAP and MTB, and due to the fact that MAP is associated with CD but not UC, we stipulated that the effect of CS on CD smokers might be similar to that of tuberculosis. The nicotine-rich HLE extracts induced macrophages to an anti-inflammatory response due to significant upregulation in *IL-10*. The opposite effect was observed when macrophages where treated with the nicotine-less LAMD extracts. The finding supports earlier observations which suggested that nicotine is a potent agent in tobacco [34,35]. The data clearly confirm that the anti-inflammatory effect observed in CS is due to nicotine. We were astonished as to how macrophages infected with MAP, or MTB, or even treated with LPS responded to exposure to nicotine. We anticipated nicotine to reduce the inflammatory response caused by the bacterial infection or the LPS derived from AIEC. Instead, the inflammatory response increased as indicated by the upregulation of *iNOS, IL-6,* and *TNF-α*. On the other hand, *CD206* and *IL-10* were downregulated (Figure 4D,E). The effect of nicotine, the most active and potent ingredient in CS, on infected macrophages is clearly opposite to that seen in uninfected macrophages. In absence of infection, nicotine acted as an immunosuppressive drug, increasing M2 macrophages and IL-10 cytokine (Figure 2); however, upon MAP infection, the anti-inflammatory role of nicotine was lost to a robust pro-inflammatory response even higher than that observed with infection only (Figure 4). This data is reflective of what is observed in IBD patients. 

Knowing that some CD patients might be suffering from infection with MAP, AIEC, or possibly other microorganisms, which is not the case in UC patients, we propose that the successive inflammatory response among some CD smokers is due to bacterial infection associated with the disease. Dysbiosis and microbiota shifts have been reported in CD patients, which lead to an alteration in the proportion of bacteroides, firmicutes, and enterobacteriaceae [14]. In this study, we included MTB as a positive control in order to confirm the expected effect of nicotine on macrophages in TB patients. We also included LPS in order to investigate the effect of gram-negative bacteria, including AIEC and *K. pneumoniae* that also have been associated with CD pathogens. The data clearly confirm that the successive inflammatory response observed in CD smokers is related to the association of the disease with bacterial infection.

Unlike the common knowledge, also confirmed in our study, about the anti-inflammatory effect of nicotine, the true effect of nicotine on macrophages depends greatly on the presence or absence of infection. A lack of infection in patients with UC should explain why they benefit from CS exposure as reported extensively in the literature [35]. In fact, nicotine is currently offered as a therapeutic agent for UC; present in many forms, such as nicotine patches, chewing gum, as well as nicotine-based enemas, because of its anti-inflammatory proprieties [36,37]. The opposite is true in CD smokers who often suffer from bacterial infection. 

Further evidence to support the outcome of this study is the use of MTB as a control. The effect of nicotine or HLE extracts on MTB infection was strongly similar to that of MAP infection. Data in our study strongly support published reports indicating TB smokers to suffer greatly from increased symptoms compared to TB non-smokers [38]. We cannot exclude that the presence of oxidative and highly reactive compounds other than nicotine in CS may contribute to the successive pro-inflammatory response observed in infected macrophages [39]. 

To confirm and validate our conclusion, we proceeded to determine the mechanism by which nicotine modulates macrophages. First, nicotine in this study increased the viability of MAP in THP-1 cells (Table 1). This confirms earlier reports from MTB studies which demonstrated that nicotine impairs anti-MTB defense within macrophages through α7nAchR, by decreasing apoptosis via activation of the NF-ҝB family—this ultimately leading to an increased MTB burden, thereof, worsening TB symptoms [33,38]. Among the virulence factors in Mycobacteria that are involved in their survival and persistence include Protein Kinase G (PknG) (an enzyme which inhibits phagosome–lysosome fusion) and Protein Tyrosine Phosphatase-A (PTP-A, an enzyme that inhibits phagosomal maturation) [40]. Based on the close relatedness between MTB and the fact that MAP data in this study are similar to MTB, we propose that MAP survival in macrophages follows a mechanism similar seen in MTB. On the other hand, the viability of *K. pneumoniae* in macrophages was poor and most likely due to the lack of these key virulence factors. Clearly, the impact of increase in MAP and MTB viability in infected macrophages has caused these immune cells to continuously and exponentially release pro-inflammatory cytokines, including IL-6 and TNF-α, as demonstrated in this study (Figure 4). Interestingly, we were able to link the upregulation in these cytokines to the polarization of macrophages into M1 as demonstrated by upregulation of *iNOS* marker. Second, we observed a significant decrease in macrophages apoptosis upon nicotine treatment, which further supports the idea that nicotine increases mycobacterial viability by reducing the host’s cell death (Figure 7). Third, nicotine exerts its immunosuppressive effect via α7nAChRs on macrophages and other immune cells. These receptors are responsible for the activation of cholinergic anti-inflammatory pathway leading to decreased secretion of pro-inflammatory cytokines, such as IL-6 and TNF-α in vitro and in vivo which confirms earlier reports [41]. Our study confirmed the immunosuppressive effect of pure nicotine in THP-1 cells by shifting the polarization to alternative M2 macrophages and increased the production of anti-inflammatory cytokine IL-10. Our study further established that abrogation of nicotine immune modulatory effect depends on the blockage of its receptor with MEC, a pharmacological inhibitor to nAchRs (Figure 3). This data support an earlier report regarding the downregulation of α7nAChRs expression in inflamed CD tissues [32].

Moreover, we investigated whether pre-conditioning macrophages with nicotine would confer protection against infection. Our results indicate that pretreatment of infected macrophages with nicotine significantly inhibits the production of pro-inflammatory cytokines (Figure 6B,C). Similar findings were reported by a previous study showing that nicotine pretreatment significantly decreased IL-6, TNF-α, and IL-12 in *Legionella* infected macrophages [42]. In addition, pre-exposing cells to nicotine induces M2 polarization, which may not be canceled or reversed back to M1 status following infection (Figure 6). We plan to investigate the ability of M2 to reverse to M1 and vice versa.

One limitation of this study is that it is an in vitro culture system in which THP-1 cells, an immortalized monocyte-like cell line, was used to mimic CD-like macrophages and perhaps peritoneal macrophages. We plan to further validate our findings in cells from CD and UC patients including peritoneal macrophages and peripheral mononuclear cells. Additionally, using an in vitro model system as in this study may suppress the true effect occurring in vivo. Future studies should involve investigations of the effect of nicotine and CS and possibly vaping in animals infected with CD-associated pathogens. 

**Figure 8:**
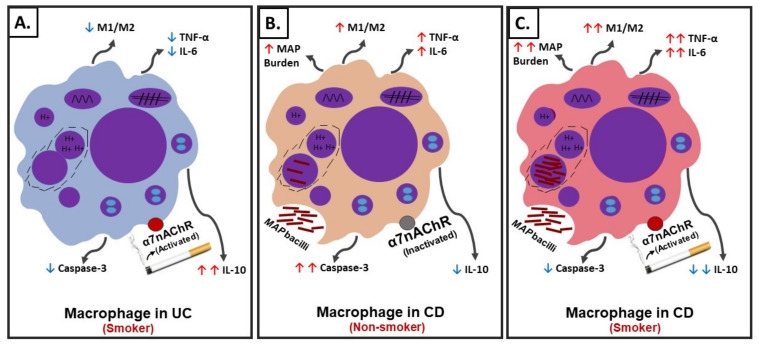
Schematic illustration of the role of cigarette smoke (CS) and nicotine in IBD. (**A**) Interaction of nicotine with receptor and cellular changes and inflammatory response in absence of infection in ulcerative colitis (UC)-like macrophages. (**B**) Interaction of nicotine with receptor and cellular changes and inflammatory response in CD-like macrophages with bacterial infection and no history of smoking. (**C**) Interaction of nicotine with receptor and cellular changes and inflammatory response in CD-like macrophages with bacterial infection and active history of smoking.

In summary, using an in vitro cell culture system, we were able to mimic a macrophages setup similar to those in CD and UC patients with history of prior or active cigarette smoking and in correlation with active bacterial infection. The effect of CS on the cellular processes in macrophages similar to those in UC active smokers, CD active smokers, and CD non-smokers are illustrated in Figure 8. Nicotine in UC active smokers activates the cholinergic anti-inflammatory pathway through α7nAChRs leading to M2-macrophage polarization, upregulation of *CD206* and *IL-10*, downregulation of *iNOS, IL-6*, and *TNF-α*, and significantly decreases caspase-3 activity (Figure 8A). Similar cellular changes are expected in CD active smokers who are not associated with infection (Figure 8A). On the contrary, nicotine in CD active smokers associated with bacterial infection induce M1-macrophage polarization, upregulation of *iNOS, IL-6*, and *TNF-α*, downregulation in *CD206*, *IL-10*, decrease in caspase-3 activity, and increase in infection burden (Figure 8C). CD non-smokers with bacterial infection suffer also from pro-inflammatory response but at a lower level (Figure 8B) compared to CD smokers with bacterial infection (Figure 8C). Cellular apoptosis is always reduced in macrophages exposed to nicotine.

Overall, this is the first study to provide significant insight toward understanding the cellular events involved in the mysterious contradictory effect of CS among IBD patients with different history of smoking. We plan to investigate the effect of CS ingredients on the molecular processes involved in bacterial infection and interaction with nicotine receptors in macrophages mimicking that of IBD patients. 

## Figures and Tables

**Table 1:** Effect of nicotine on bacterial viability in macrophages.

Treatment Group	Viability Mean ± SD (%)	Fold Change in Viability Prior/Post Nicotine Treatment
*K. pneumoniae* ATCC 13883	3.7 ± 4.5	
MAP UCF4	69.4 ± 5.2	
N(4 µg/mL) + MAP UCF4	91.4 ± 5.5	1.31
MAP UCF4 + N(4 µg/mL)	80.79 ± 4.8	1.17
MTB HR237	74.2 ± 3.1	
N(4 µg/mL) + MTB HR237	92.5 ± 4.6	1.25
MTB HR237 + N(4 µg/mL)	85.2 ± 6.2	1.15

N: nicotine. MAP: mycobacterium avium paratuberculosis. MTB: mycobacterium tuberculosis.
